# Toward robust AI-based ECG R-peak detection for real-time physiological monitoring in operational environments

**DOI:** 10.3389/fpsyg.2026.1892662

**Published:** 2026-08-06

**Authors:** Maja Boström, Erik Jonsäll, Fredrik Allenmark, Olivier Amoignon, Erik Persson, Guilherme H. Elcadi

**Affiliations:** 1 Division for Defence Technology, Department for Aeronautical Engineering, Swedish Defence Research Agency, Stockholm, Sweden; 2 Division for Defence Technology, Department for Military Air Systems, Swedish Defence Research Agency, Stockholm, Sweden; 3 Division for Cyber Defence and C2 Technology, Department for Cognitive AI-systems, Swedish Defence Research Agency, Stockholm, Sweden

**Keywords:** artificial intelligence, heart rate variability, operational environments, physiological monitoring, R-peak detection

## Abstract

Reliable estimation of heart rate variability (HRV) from electrocardiography (ECG) depends on accurate detection of R-peaks, a task that becomes challenging in operational environments characterized by noise, motion artifacts, and monitoring-grade sensor configurations. While classical detection algorithms perform well under controlled conditions, their robustness is often limited in realistic settings. This study investigates whether artificial intelligence (AI)-based approaches can achieve robust R-peak detection in noisy operational ECG recordings, providing a reliable foundation for downstream HRV analysis. A physiologically constrained annotation framework was developed to generate temporally consistent reference annotations for evaluation of operational ECG recordings, while model training was performed exclusively using the MIT-BIH Arrhythmia Database with noise augmentation from the MIT-BIH Noise Stress Test Database. Two AI-based approaches were evaluated: a bidirectional Long Short-Term Memory (LSTM) network and a Transformer-based time-series foundation model (Mantis-8M) with task-specific output layers. Model performance was evaluated using both the MIT-BIH Arrhythmia Database and a real-world dataset comprising monitoring-grade ECG recordings from 30 participants acquired under operational conditions. The strongest-performing AI-based models achieved near-perfect benchmark performance (F1 ≈ 0.99) and maintained high accuracy under noisy conditions. On operational data, the LSTM model achieved the strongest overall performance (F1 = 0.96), substantially outperforming classical Pan–Tompkins implementations and alternative AI architectures. Additional validation analyses demonstrated consistent performance across participants, low RR interval error, and minimal variability across repeated training runs, supporting the robustness and reproducibility of the proposed approach. The results demonstrate that AI-based R-peak detection preserves physiologically consistent beat-to-beat timing in noisy, monitoring-grade ECG recordings, providing a robust foundation for downstream HRV analysis. The study focuses on robustness in operational, non-clinical environments rather than diagnostic ECG interpretation. These findings support AI-based ECG analysis, trained on physiologically valid benchmark data, as a robust and reproducible foundation for real-time physiological monitoring in complex operational environments.

## 1 Introduction

Physiological monitoring has become a central component in the assessment of human performance, cognitive state, and operational readiness in both controlled and real-world environments. In domains such as aviation, defense, and other high-demand human-machine systems, continuous assessment of operator state, including stress, workload, and fatigue, is critical for maintaining safety and optimizing performance (Brookings et al., 1996; Wilson, 2002). Among available physiological signals, electrocardiography (ECG) provides a particularly robust and widely used basis for deriving heart rate (HR) and heart rate variability (HRV), which serve as noninvasive markers of autonomic nervous system dynamics and cognitive load (Shaffer and Ginsberg, 2017; Task Force of the European Society of Cardiology and the North American Society of Pacing and Electrophysiology, 1996).

The reliability of HR and HRV metrics depends fundamentally on accurate detection of R-peaks within the ECG signal. Even small timing errors can propagate into distortions in derived HRV measures, particularly in short-term and frequency-domain analyses (Berntson et al., 1997; Clifford et al., 2006). This dependency becomes particularly critical in operational environments, where monitoring-grade ECG recordings are frequently affected by motion artifacts, amplitude variability, and nonstationary noise (Clifford et al., 2012).

Classical algorithms for R-peak detection, such as the Pan-Tompkins method, have historically demonstrated strong performance under controlled, clinical conditions (Pan and Tompkins, 1985). However, their reliance on fixed thresholds, handcrafted features, and assumptions of signal stationarity limits their robustness in noisy and heterogeneous environments (Li et al., 1995; Elgendi, 2013). As a result, their performance can degrade substantially when applied to ECG data collected in realistic operational contexts, including flight simulators, field deployments, and wearable sensing scenarios.

In contrast to classical threshold-based approaches, recent advances in artificial intelligence (AI), particularly deep learning and foundation models for time-series data, offer new opportunities to address these limitations. Recurrent neural networks such as Long Short-Term Memory (LSTM) architectures capture temporal dependencies in physiological signals (Hochreiter and Schmidhuber, 1997), while transformers in general and foundation models in particular enable scalable learning across diverse datasets (Vaswani et al., 2017; Zerveas et al., 2021; Feofanov et al., 2025). These methods have demonstrated strong potential for robust pattern recognition in complex, nonstationary data, making them promising candidates for ECG analysis in operational environments (Hannun et al., 2019; Laitala et al., 2020).

Nevertheless, a critical challenge in this context lies not only in model design, but in ensuring that learned representations reflect true physiological dynamics rather than artifact-driven signal patterns. During model development, high-quality annotations play an essential role in guiding learning toward physiologically meaningful timing relationships (Goldberger et al., 2000; Clifford et al., 2017). However, the ultimate objective of real-time physiological monitoring systems is to enable reliable, fully automated operation without dependence on manual correction or annotated reference signals.

This is especially relevant in operational environments, where continuous monitoring must be performed under conditions of variable signal quality, limited supervision, and strict performance requirements (Parasuraman and Wilson, 2008). Robust performance in these settings requires models that generalize beyond controlled datasets and maintain physiologically consistent beat-to-beat timing in the presence of noise and signal degradation.

Recent work has increasingly explored AI-based approaches for physiological monitoring under wearable and real-world conditions. For example, Thapa et al. (2025) proposed a hybrid wavelet–deep learning framework for robust ECG analysis in wearable monitoring applications, highlighting the growing importance of accurate and computationally efficient physiological signal processing. Beyond individual signal-processing algorithms, broader body-area network frameworks have emphasized the need for interoperable, low-latency sensing architectures capable of supporting continuous physiological monitoring across heterogeneous sensor systems (Hämäläinen et al., 2020). The present work complements these developments by focusing on robust R-peak detection and physiologically consistent RR interval estimation, which constitute foundational requirements for future real-time physiological monitoring systems.

This work does not aim to address clinical-grade ECG interpretation or diagnostic accuracy, but rather focuses on robust R-peak detection in monitoring-grade signals acquired under operational conditions, where noise resilience and temporal consistency are critical for downstream HRV analysis.

Within this context, the present study investigates whether AI-based R-peak detection models trained on benchmark physiological data can achieve sufficient robustness to generalize to noisy, operationally realistic ECG recordings. To this end, we combine a physiologically constrained annotation framework for monitoring-grade ECG data with the development and evaluation of advanced AI models, including LSTM-based architectures and a time-series foundation model.

**Figure 1:**
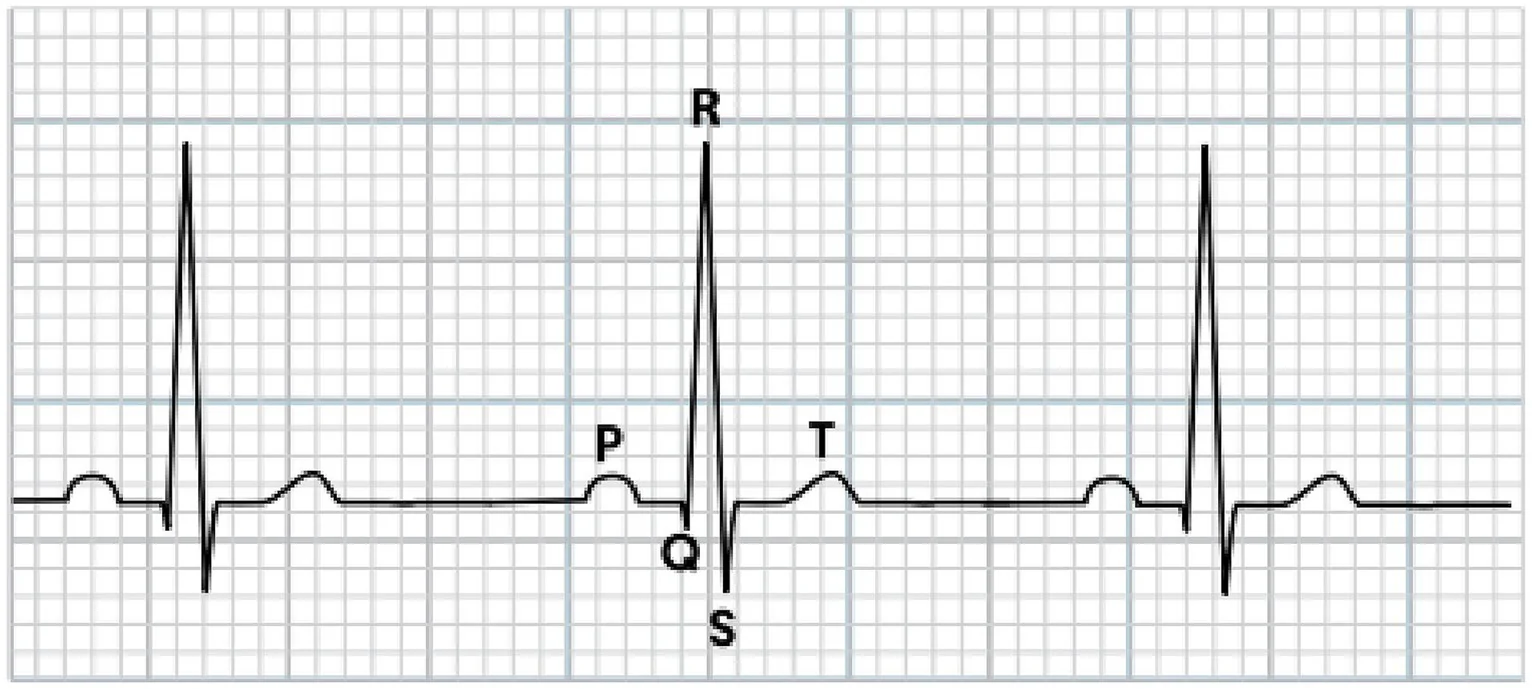
Standard ECG waveform morphology. Illustration of a typical ECG cycle showing the P wave, QRS complex, and T wave (adapted with permission from Ergun_Pinar/Shutterstock.com). The R-peak corresponds to the maximum amplitude within the QRS complex and is used for beat-to-beat timing and HRV analysis.

Model performance is assessed using both benchmark datasets and real-world recordings, enabling evaluation under controlled as well as operational conditions. Particular emphasis is placed on maintaining accurate beat-to-beat timing under noise, as this represents a critical requirement for downstream HRV analysis. The R-peak, corresponding to the maximal deflection of the QRS complex in the ECG waveform (Figure 1), serves as the fundamental temporal marker for beat-to-beat interval estimation.

**Figure 2:**
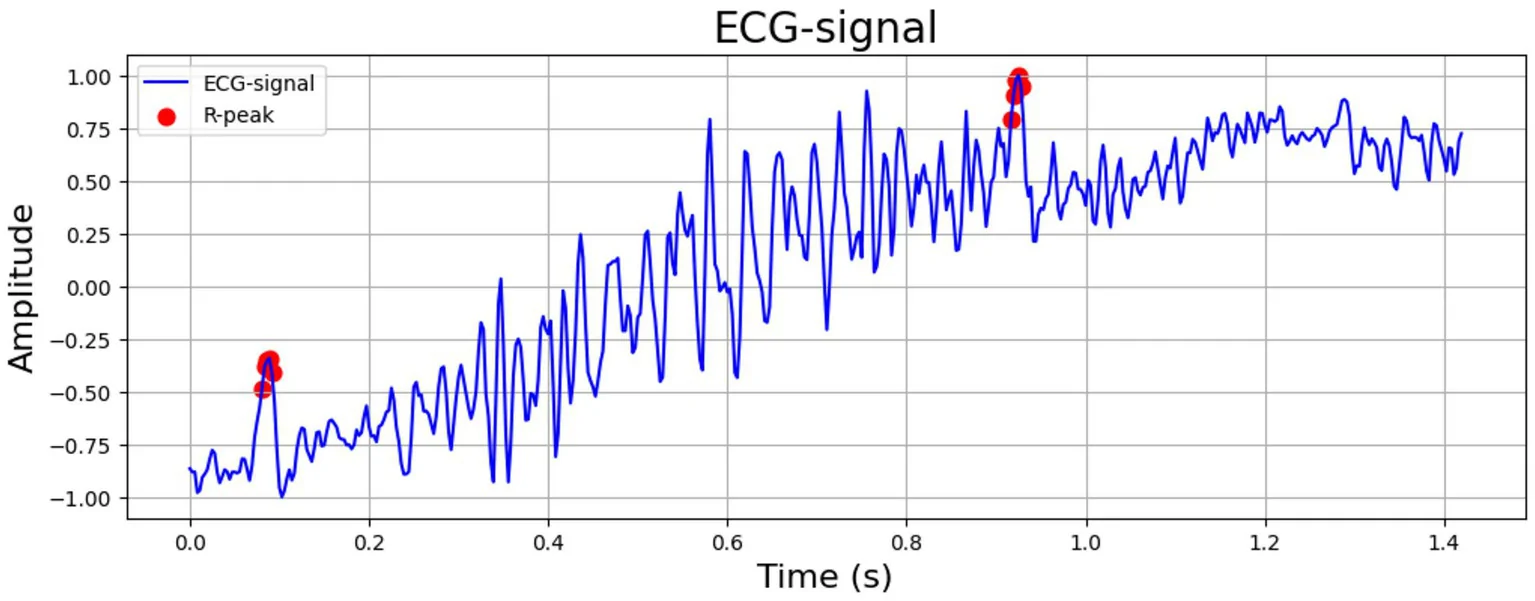
Example of noisy ECG signal illustrating the challenge of R-peak detection in operational environments. A representative segment of monitoring-grade ECG data is shown, characterized by motion artifacts, amplitude variability, and nonstationary noise. Several R-peaks are difficult to identify due to signal corruption, particularly in the central portion of the recording, highlighting the limitations of classical threshold-based detection methods under realistic conditions. Dots indicate the annotated R-peaks.

The central hypothesis underlying this work is that reliable AI-based physiological monitoring does not depend on manual correction during deployment, but rather on the ability of models to learn physiologically valid representations during training. When this condition is met, AI-based R-peak detection can provide a robust foundation for real-time HRV analysis in complex and noisy operational environments. An example of such signal degradation is shown in Figure 2.

## 2 Methods

### 2.1 Data annotation framework

**Figure 3:**
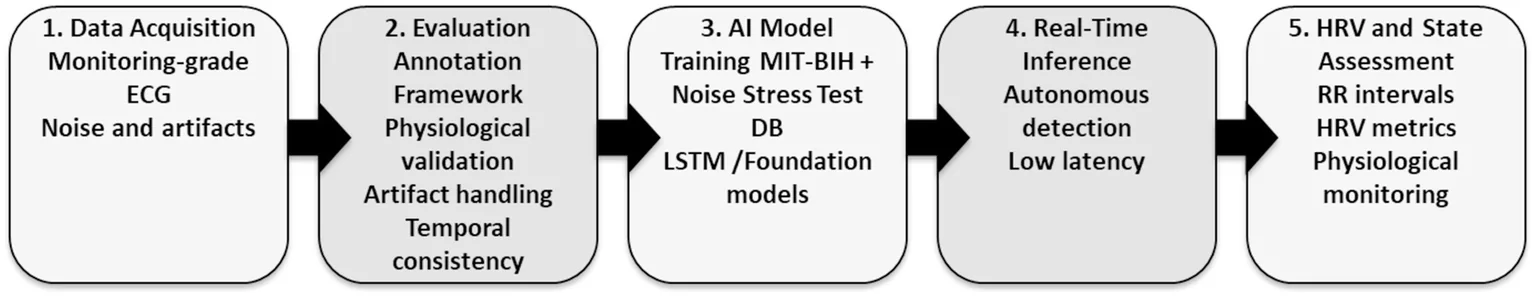
Framework for AI-based real-time physiological monitoring using ECG. The figure illustrates the end-to-end pipeline for robust R-peak detection and HRV analysis in operational environments. Monitoring-grade ECG signals are acquired under realistic conditions characterized by noise and motion artifacts. During evaluation of operational ECG data, physiologically consistent annotations are generated to preserve cardiac timing while handling artifacts conservatively. AI-based models, including LSTM and foundation-model architectures, are trained using benchmark ECG data and subsequently evaluated under operational conditions. While the proposed architecture is designed to support future autonomous, real-time operation for R-peak detection without manual intervention, the present study focuses on the development and evaluation framework, including data acquisition, annotation methodology, and AI-based model training and evaluation. Fully autonomous deployment and downstream physiological state assessment therefore remain subjects for future work.

To support the development and evaluation of AI-based R-peak detection models under realistic conditions, a physiologically constrained annotation framework was established for monitoring-grade ECG recordings. The primary objective of this framework was to generate reference annotations that preserve true cardiac timing while remaining robust to noise and artifacts commonly encountered in operational environments. An overview of the proposed development and deployment pipeline is presented in Figure 3.

ECG data were acquired using a two-electrode bipolar chest configuration and treated as monitoring-grade signals, with priority given to beat-to-beat timing over diagnostic waveform interpretation. In contrast to clinical ECG, such recordings exhibit substantial variability in amplitude, morphology, and signal quality, including motion artifacts, electrode disturbances, and transient nonstationary noise.

To ensure the integrity of R-peak annotations, preprocessing and validation during model development were guided by three core principles, described in more detail below:

(i) Preservation of the original signal time base,
(ii) Enforcement of physiological plausibility constraints, and
(iii) Conservative handling of ambiguous signal segments, whereby uncertain detections are avoided unless supported by clear physiological and temporal evidence.

Importantly, all corrections were restricted to R-peak annotations. The ECG signal itself was never resampled, truncated, or temporally modified.

First, initial R-peak detection was performed using a sensitive amplitude-based approach with a minimum inter-peak distance corresponding to a maximum physiological heart rate of 200 beats min^−1^. Subsequently, validation addressed non-physiological spike artifacts using a combination of amplitude and derivative-based criteria, enabling identification of unrealistically steep and high-amplitude signal excursions. To prevent non-physiological transients from being interpreted as cardiac events, detections occurring within a short blanking interval surrounding the artifact (20 ms before and 60 ms after the detected artifact) were removed. Potentially missed beats were subsequently reacquired only when candidate peaks satisfied predefined physiological timing constraints, including minimum RR interval and refractory-period requirements consistent with plausible cardiac rhythms.

Importantly, in segments characterized by sustained artifact activity, automated correction was deliberately suspended to avoid introducing physiologically implausible timing. These segments were retained for evaluation but excluded from automated correction procedures to preserve data integrity.

All annotations of the operational ECG recordings were performed by a single researcher with extensive experience in ECG processing, R-peak identification, HRV analysis, and advanced medical training. Annotation decisions followed the physiologically informed framework described above, with particular emphasis on preserving beat-to-beat timing while handling noise and artifacts conservatively. Potential artifact-related detections were reviewed manually, physiologically implausible detections were rejected, and clustered detections were consolidated into a single representative R-peak. Because the annotation process was performed by a single expert annotator, inter-rater agreement statistics and disagreement-resolution procedures were not applicable.

The resulting R-peak annotations were subsequently reviewed manually to ensure consistency and physiological plausibility before being accepted as reference annotations for evaluation of operational ECG recordings. This annotation process was applied exclusively during the development phase and therefore does not constitute a requirement for operational deployment.

Accurate R-peak timing is essential for model training. In this study, physiologically consistent annotations were generated using expert-informed and rule-based procedures to ensure temporal validity. While such approaches may involve human oversight, the critical requirement is the preservation of physiologically valid beat-to-beat timing. The annotation framework was used to establish physiologically consistent reference annotations for evaluation of operational ECG recordings. AI model training, however, was performed exclusively using the MIT-BIH Arrhythmia Database.

### 2.2 MIT-BIH arrhythmia database

A publicly available benchmark dataset, the MIT-BIH Arrhythmia Database (Goldberger et al., 2000), was employed for initial model training and validation. The dataset consists of 47 participants, partitioned into 30 for training, 10 for validation, and 7 for testing. The dataset provides annotated ECG recordings sampled at 360 Hz, with expert-labeled R-peaks. During preprocessing, clustered labels were generated around each annotated R-peak following the strategy described by Laitala et al. (2020) to improve training stability. During evaluation, clustered predictions were reduced to a single representative R-peak by selecting the maximum amplitude within each detected region.

**Figure 4:**
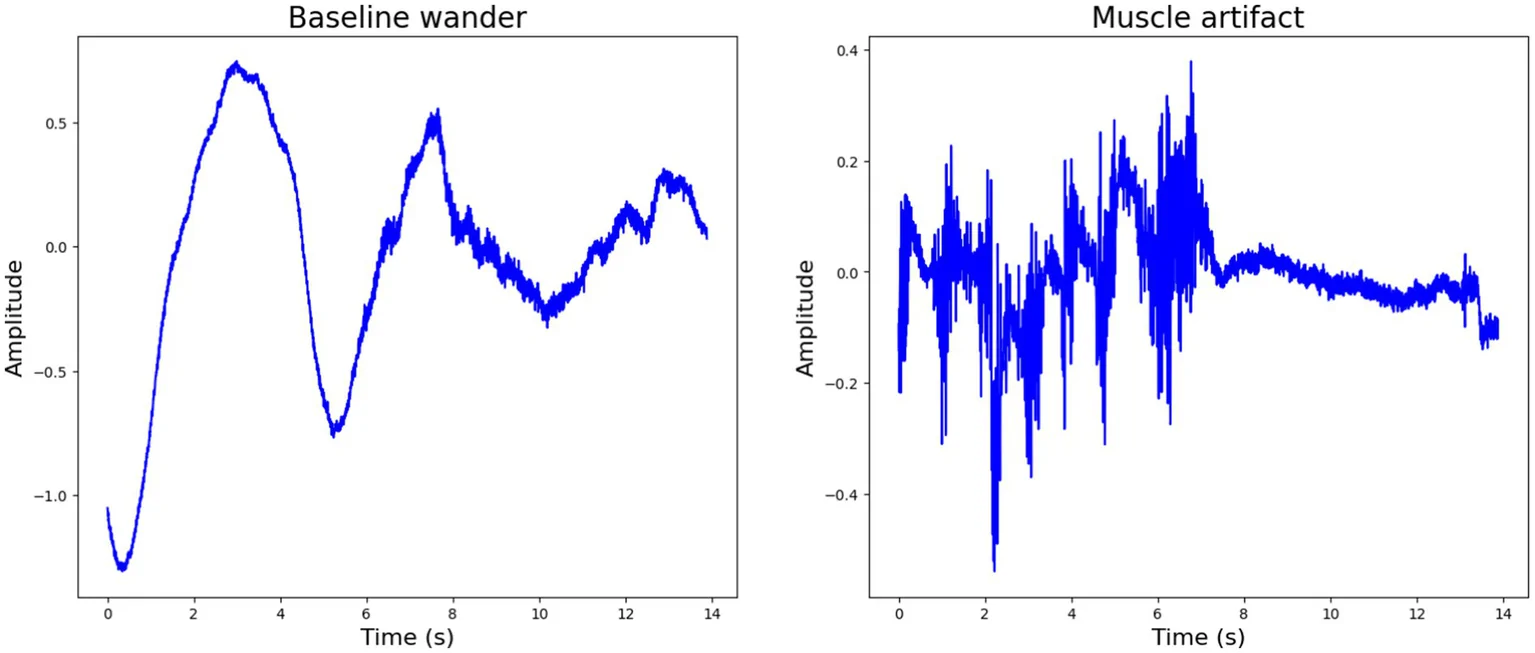
Examples of common noise artifacts in monitoring-grade ECG signals. Representative ECG segments illustrating two typical sources of noise encountered in operational environments. The left panel shows baseline wander, characterized by low-frequency drift often caused by respiration, movement, or electrode instability. The right panel shows muscle artifact, characterized by high-frequency irregular fluctuations resulting from muscle activity. Both types of noise can distort ECG morphology and complicate accurate R-peak detection in monitoring-grade recordings acquired under non-controlled conditions.

To assess robustness to noise, synthetic noise components from the MIT-BIH Noise Stress Test Database (Moody et al., 1984) were incorporated during model development, enabling controlled evaluation under varying signal-to-noise conditions. Representative examples of noise types, baseline wander and muscle artifact, relevant to monitoring-grade ECG are shown in Figure 4.

### 2.3 Swedish Defence Research Agency (FOI) operational dataset

To evaluate real-world performance, a dataset consisting of ECG recordings from 30 healthy participants (25 males, 5 females; mean age approximately 34 years) was used. The recordings originated from a previously conducted virtual reality study investigating simulator sickness during repeated roller-coaster simulator exposures (Elcadi et al., 2026). Participants were exposed to a realistic roller-coaster simulation using a head-mounted display while continuous ECG recordings were acquired. The protocol consisted of a 5-min baseline period followed by up to five roller-coaster rides of approximately 2.5 min each. The resulting recordings were characterized by substantial physiological variability and motion-related artifacts associated with realistic VR exposure, providing a challenging monitoring-grade evaluation condition. These recordings were obtained using monitoring-grade sensor configurations and exhibit realistic levels of noise, motion artifacts, and signal variability representative of operational physiological monitoring environments.

For model evaluation, the FOI ECG recordings were downsampled from 2000 Hz to 360 Hz to match the sampling frequency of the MIT-BIH dataset and ensure compatibility across training conditions. No additional signal modification was applied during this preprocessing step. The downsampling procedure was used exclusively for model evaluation and was not part of the annotation process.

### 2.4 AI-based R-peak detection models

The central focus of this study is the evaluation of AI-based approaches for robust R-peak detection under noisy and heterogeneous conditions. Two classes of models were investigated: recurrent neural networks and a transformer-based foundation model for time-series analysis.

The AI-based models were trained exclusively using the MIT-BIH Arrhythmia Database together with noise augmentation derived from the MIT-BIH Noise Stress Test Database. The FOI operational dataset was not used during model training and was instead reserved for inference and evaluation under realistic operational conditions.

#### 2.4.1 LSTM-based model

A Long Short-Term Memory (LSTM) network was implemented to capture temporal dependencies in ECG signals. The architecture comprises two bidirectional LSTM layers, each containing 64 cells, followed by a dense output layer enabling sequence-to-sequence prediction of R-peak locations. Input sequences of fixed length (512 samples) were used, and the model outputs a binary mask indicating the presence of R-peaks at each time step.

**Figure 5:**
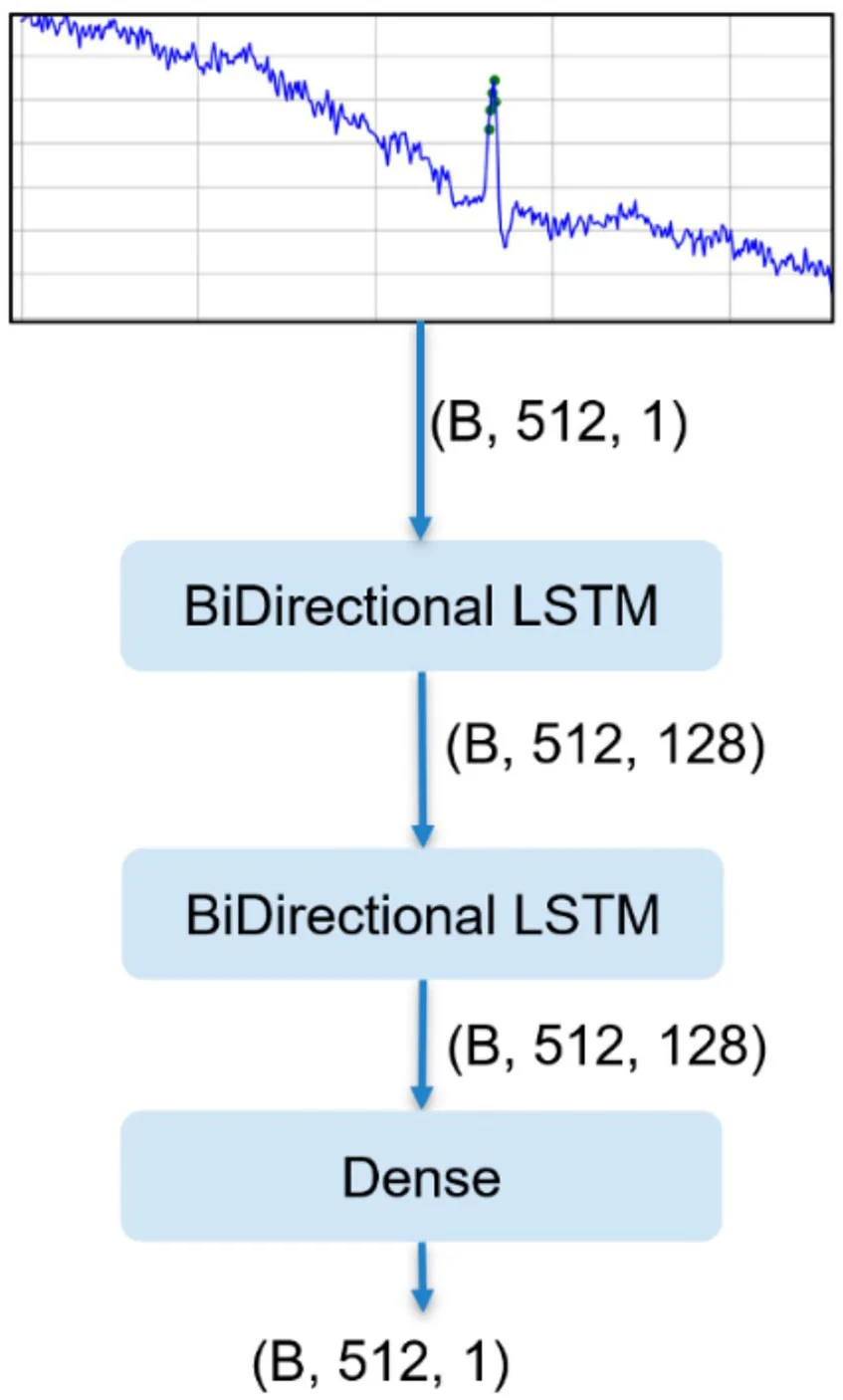
LSTM-based architecture for R-peak detection. The model processes fixed-length ECG segments and outputs a binary sequence indicating the presence of R-peaks at each time step. Bidirectional LSTM layers capture temporal dependencies across cardiac cycles, enabling discrimination between physiological R-peaks and noise-related artifacts. The architecture is based on the R-peak detection approach described by Laitala et al. (2020). Tensor dimensions are indicated for each stage, where *B* denotes batch size.

The LSTM architecture is particularly suited for ECG analysis due to its ability to retain temporal context across multiple cardiac cycles, allowing it to distinguish true physiological patterns from transient noise. The LSTM-based architecture used for R-peak detection is shown in Figure 5.

#### 2.4.2 Foundation model (Mantis-8M)

To explore the potential of large-scale pretrained models, a foundation model for time-series classification (Mantis-8M) was evaluated. This model is based on a transformer-inspired architecture and has been pretrained on a large and diverse set of time-series data, including physiological signals.

**Figure 6:**
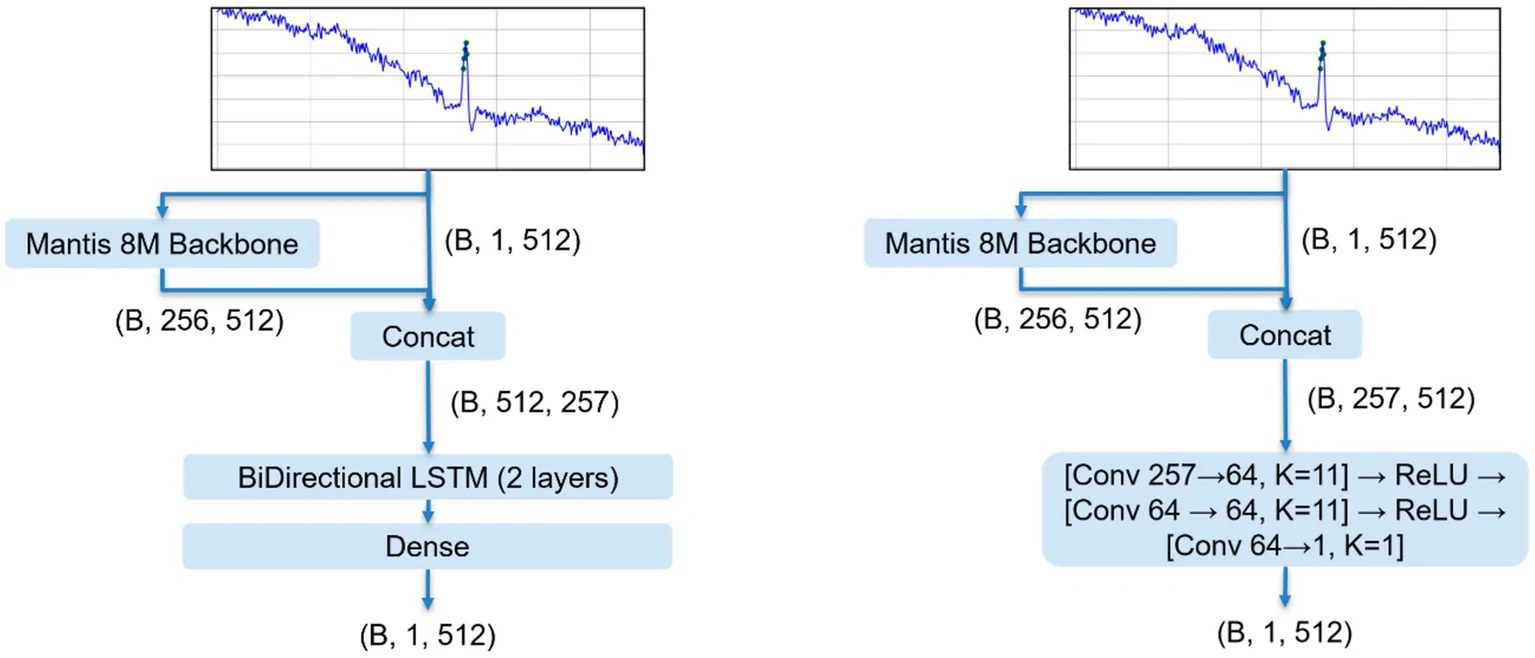
Architectures of the Mantis-8M-based R-peak detection models with task-specific output layers. Two variants of the foundation model approach are illustrated. In both cases, input ECG segments are first processed by the pretrained Mantis-8M backbone, which extracts high-dimensional feature representations. These features are concatenated with the original input signal to preserve temporal alignment and signal-level information. Left panel: Mantis-8M + LSTM architecture. The concatenated features are passed to a bidirectional LSTM network (two layers), which captures temporal dependencies across cardiac cycles before a dense layer produces a binary R-peak prediction sequence. Right panel: Mantis-8M + CNN architecture. The concatenated features are processed by a series of convolutional layers with ReLU activations, progressively reducing the feature dimension and mapping the representation to a single-channel output corresponding to R-peak predictions. Tensor dimensions are indicated for each stage, where *B* denotes batch size and the final output corresponds to a binary sequence indicating the presence of R-peaks at each time step.

As the base model produces feature representations rather than direct R-peak predictions, additional layers were introduced to map extracted features to a binary R-peak detection output. Two variants were evaluated: a Mantis-8M model with a convolutional neural network (CNN) head and a Mantis-8M model with an LSTM head. The two Mantis-8M-based model configurations are illustrated in Figure 6. This approach enables leveraging pretrained representations while adapting the model to the specific task of R-peak detection.

### 2.5 Baseline method

For comparison, two implementations of the classical Pan–Tompkins algorithm were evaluated: the Python-based implementation available in the py-ecg-detectors package and a MATLAB implementation available through MATLAB central^1^^,^^2^. These implementations reflect the variability commonly encountered in practical use, where algorithm performance depends on parameter tuning and implementation details. Evaluating multiple variants provides a more realistic baseline for comparison with AI-based methods.

### 2.6 Input representation and windowing

ECG signals were segmented into fixed-length windows prior to model input. A window length of 512 samples was selected to balance temporal context and computational efficiency, while ensuring compatibility with the foundation model architecture, which requires input lengths that are multiples of 32.

All models were trained on fixed-length segments derived from the MIT-BIH dataset together with noise-augmented training data and subsequently evaluated on both benchmark and operational ECG recordings.

### 2.7 Evaluation protocol

Model performance was evaluated by comparing predicted R-peaks with reference annotations using standard classification metrics defined in (Hicks et al., 2022), including Precision, Recall, and F1-score, based on a single inference run.

To account for minor temporal deviations, a tolerance window of ±10 ms (corresponding to ±4 samples at 360 Hz) was applied. This threshold was selected to accommodate small temporal uncertainties associated with both reference annotations and automated detections while remaining substantially smaller than physiologically meaningful RR interval variations. A predicted peak was considered correct if it fell within this tolerance range of a reference R-peak. The selected tolerance therefore provides a conservative criterion for assessing temporal accuracy and is considerably more stringent than evaluation tolerances commonly used in ECG beat-detection studies (e.g., Laitala et al., 2020), while avoiding undue penalization of detections exhibiting only minimal timing differences.

To ensure fair comparison across methods, reference annotations were reduced to a single representative R-peak per cardiac cycle, and all models were evaluated using the same tolerance criteria. This evaluation framework is used solely to quantify model performance relative to reference annotations and does not reflect a requirement for annotated input during deployment.

The clustered annotation structure originates from the preprocessing strategy employed in the LSTM-based reference implementation (Laitala et al., 2020), where groups of neighboring samples are labeled around each R-peak to improve training stability. Consequently, model outputs may also form clustered predictions, which were reduced to a single representative peak during evaluation to enable fair comparison with classical detection algorithms.

### 2.8 Real-time implementation considerations

The evaluated models are designed for real-time applicability in physiological monitoring systems. Input segmentation, model architectures, and output representations were selected to support sequential processing and low-latency inference.

Importantly, while reference annotations are required during training and evaluation, the deployed models will operate fully autonomously, enabling real-time R-peak detection without manual intervention or access to annotated signals.

## 3 Results

### 3.1 Performance on benchmark data

As an initial step, the performance of R-peak detection methods was evaluated on the MIT-BIH Arrhythmia Database under both clean and noise-augmented conditions. As summarized in Table 1, the strongest-performing AI-based models demonstrated near-perfect performance on clean data, with both the LSTM and Mantis-8M-based approaches achieving F1-scores close to 1.0. In contrast, the classical Pan-Tompkins algorithm exhibited lower performance, particularly in precision, reflecting its sensitivity to signal variability.

**Table 1:** Performance comparison of R-peak detection methods across benchmark and operational datasets.

Method	MIT-BIH (clean) F1	MIT-BIH (noise) F1	FOI dataset F1	Precision (FOI)	Recall (FOI)
Pan–Tompkins v1	0.78	0.72	0.65	0.53	0.94
Pan–Tompkins v2	0.08	0.12	0.07	0.07	0.07
LSTM	**0.99**	**0.98**	**0.96**	**0.94**	**0.99**
Mantis-8M + CNN	0.98	0.94	0.84	0.81	0.87
Mantis-8M + LSTM	**0.99**	0.97	0.30	0.18	0.79

Results are reported in terms of F1-score, precision, and recall, based on a single inference run. AI-based models were trained using the MIT-BIH Arrhythmia Database together with noise augmentation derived from the MIT-BIH Noise Stress Test Database and subsequently evaluated on benchmark and operational ECG recordings. The LSTM-based and Mantis-8M + CNN models demonstrated robust performance across noisy and operational conditions, whereas performance degradation observed in certain configurations (e.g., Mantis-8M + LSTM on FOI data) highlights the importance of domain alignment and downstream adaptation strategies. Bold values indicate the highest performance within each evaluation metric.

However, under noise-augmented conditions, the performance of classical methods remained substantially worse than that of the strongest-performing AI-based models. In contrast, the strongest-performing AI-based models maintained high detection accuracy, with only a modest reduction in F1-score. This indicates that learned representations are robust to nonstationary noise and artifact contamination, preserving accurate identification of R-peak locations under degraded signal conditions. These results establish a strong performance baseline under controlled conditions, against which robustness in operational data can be interpreted.

### 3.2 Performance on operational data

To assess performance under realistic conditions, models were evaluated on the FOI dataset, consisting of monitoring-grade ECG recordings acquired in operationally relevant environments. As shown in Table 1, the LSTM-based and Mantis-8M + CNN approaches maintained high performance despite the presence of motion artifacts, amplitude variability, and nonstationary noise. The FOI dataset, which was reserved exclusively for evaluation and not included during training, provided a challenging operational test condition characterized by substantial noise and signal variability.

Notably, the LSTM model achieved the highest overall performance on this dataset, combining high precision with near-perfect recall. The Mantis-8M-based model with a convolutional head also demonstrated robust performance, although with some reduction in precision compared to the LSTM. In contrast, the classical Pan-Tompkins algorithm showed a substantial decrease in precision, indicating a high rate of false detections in noisy conditions.

Importantly, the combination of high recall and strong precision observed in the LSTM-based and Mantis-8M + CNN models indicates that both missed beats and spurious detections are minimized. This is critical for preserving the temporal integrity of inter-beat intervals, supporting reliable HRV computation under operational conditions. The results therefore suggest that the detected R-peak sequences maintain physiologically consistent beat-to-beat timing even under operational noise conditions.

### 3.3 Model comparison

Across all datasets, the LSTM-based model consistently achieved the most balanced and highest overall performance, maintaining both high precision and recall under controlled and operational conditions. This suggests that temporal modeling of sequential dependencies plays a critical role in distinguishing true R-peaks from artifact-driven signal fluctuations.

The foundation model (Mantis-8M) exhibited strong performance on benchmark data and moderate robustness under noise, particularly when combined with a convolutional output layer. However, performance varied across configurations, with the Mantis-8M + LSTM variant showing reduced precision on the FOI dataset. This indicates that, while pretrained representations can support robust detection, their effectiveness depends on appropriate adaptation to the target domain.

Overall, the LSTM-based and Mantis-8M + CNN models substantially outperformed classical methods across most evaluation conditions. The consistency of these results across both benchmark and operational datasets demonstrates that accurate and stable R-peak detection can be achieved in monitoring-grade ECG recordings without reliance on controlled acquisition conditions.

### 3.4 Statistical and operational validation

Additional validation analyses were performed to further characterize the robustness and practical applicability of the evaluated models. Table 2 presents 95% confidence intervals (CI) for the F1-scores of each evaluated method, calculated using Student’s t-distribution based on the mean and standard deviation across the evaluated noise levels, thereby providing an estimate of the statistical uncertainty associated with the reported performance. The LSTM model exhibited the highest F1-score together with a narrow CI, indicating stable detection performance across the evaluation dataset.

**Table 2:** F1-scores and 95% CIs calculated using Student’s *t*-distribution for R-peak detection methods evaluated on the FOI dataset.

Method	F1-score (FOI)	CI 95% (FOI)
Pan–Tompkins v1	0.65	0.61–0.68
Pan–Tompkins v2	0.07	0.06–0.09
LSTM	**0.96**	**0.93–0.98**
Mantis-8M + CNN	0.84	0.74–0.89
Mantis-8M + LSTM	0.30	0.25–0.36

The CIs provide a measure of the statistical precision for each method’s F1-score. The results indicate that LSTM and Mantis-8M + CNN demonstrate high precision and stability, whereas both Pan-Tompkins versions along with Mantis-8M + LSTM show significant lowering in performance. Bold values indicate the highest F1-score and its corresponding 95% confidence interval.

To assess subject-wise variability, Table 3 summarizes detection performance for each individual participant together with the corresponding signal-to-noise ratio (SNR). Although all evaluated methods generally benefited from improved signal quality, the LSTM consistently maintained high detection performance across subjects, demonstrating greater robustness under the varying recording conditions characteristic of monitoring-grade operational ECG.

**Table 3:** Per-subject F1-scores for each evaluated R-peak detection method together with the estimated signal-to-noise ratio (SNR).

Subject	F1-score LSTM	F1-score Mantis 8M + LSTM	F1-score Mantis 8M + CNN	F1-score PT1	F1-score PT2	SNR [dB]
1	0.94	0.32	0.83	0.63	0.04	11
2	0.99	0.34	0.96	0.82	0.07	14
3	0.98	0.29	0.93	0.63	0.08	14
4	0.97	0.69	0.93	0.65	0.07	15
5	0.95	0.36	0.87	0.71	0.18	13
6	0.93	0.17	0.76	0.54	0.18	7
7	0.73	0.16	0.52	0.42	0.05	3
8	0.98	0.35	0.93	0.62	0.02	16
9	0.82	0.16	0.25	0.45	0.11	-3
10	0.93	0.28	0.82	0.67	0.09	10
11	0.90	0.24	0.66	0.54	0.04	3
12	1.00	0.59	0.99	0.75	0.06	16
13	0.96	0.28	0.65	0.57	0.08	10
14	1.00	0.34	0.94	0.64	0.07	11
15	1.00	0.21	0.93	0.72	0.02	16
16	0.99	0.46	0.57	0.74	0.10	14
17	0.99	0.17	0.98	0.76	0.03	15
18	0.99	0.40	0.97	0.77	0.02	16
19	0.92	0.41	0.91	0.58	0.05	12
20	1.00	0.59	0.99	0.78	0.05	16
21	0.99	0.10	0.97	0.60	0.02	15
22	0.99	0.29	0.99	0.80	0.05	13
23	0.93	0.33	0.71	0.55	0.06	5
24	0.97	0.18	0.25	0.61	0.05	6
25	0.97	0.44	0.95	0.59	0.06	15
26	0.98	0.13	0.67	0.57	0.15	6
27	0.98	0.14	0.94	0.71	0.08	14
28	0.98	0.10	0.92	0.59	0.11	15
29	0.92	0.42	0.81	0.64	0.09	11
30	0.99	0.33	0.94	0.70	0.07	14

The table illustrates subject-wise variability and demonstrates the influence of signal quality on detection performance. Although all methods generally exhibit improved performance with increasing SNR, the LSTM model maintains comparatively higher robustness under noisier recording conditions than the remaining approaches.

Finally, Table 4 reports the mean absolute error (MAE) of the derived RR interval series relative to the reference annotations. The LSTM achieved the lowest RR interval error among all evaluated methods, indicating superior preservation of beat-to-beat timing. This finding further supports the suitability of the proposed approach for downstream physiological analyses that depend on accurate RR interval estimation, including heart rate variability assessment.

**Table 4:** Comparison of the mean absolute error (MAE) of the estimated RR-intervals with standard deviation against FOI ground truth for all evaluated methods.

Method	MAE RR [ms]	± SD [ms]
Pan–Tompkins v1	423	146
Pan–Tompkins v2	152	130
LSTM	**61**	**99**
Mantis-8M + CNN	166	175
Mantis-8M + LSTM	621	204

The Pan-Tompkins algorithms along with Mantis-8M + LSTM shows significant deviations preserving the temporal structure of the RR interval series. Bold values indicate the lowest mean absolute RR interval error and its corresponding standard deviation.

### 3.5 Computational performance and reproducibility

In addition to detection accuracy, practical deployment of AI-based ECG analysis in operational environments requires computational efficiency and reproducible model performance. To evaluate these aspects, inference times were measured for both complete dataset processing and single-frame inference using standardized hardware, with the corresponding results presented in Tables 5 and 6. Furthermore, reproducibility was assessed by retraining the LSTM model using multiple random initialization seeds while maintaining identical training data and model configuration. Together, these analyses provide additional evidence regarding the computational feasibility and stability of the proposed framework for real-time physiological monitoring.

**Table 5:** Mean prediction time (± SD) required for each evaluated method to process the complete FOI operational ECG dataset using a 13th Gen Intel® Core™ i5-1335U CPU.

Method	FOI data Mean time prediction [s]	FOI data ± SD [s]
LSTM	**112.82**	**0.943**
Mantis-8M + CNN	174.44	1.092
Mantis-8M + LSTM	212.50	2.053

Statistics were calculated from 10 independent inference runs. The LSTM model exhibited the shortest mean processing time for complete dataset inference, demonstrating computational efficiency for large-scale operational ECG analysis. Bold values indicate the shortest mean processing time and its corresponding standard deviation.

**Table 6:** Mean prediction time (± SD) required for each evaluated method to perform inference on a single ECG time frame using a 13th Gen Intel® Core™ i5-1335U CPU.

Method	FOI data Mean time prediction [s]	FOI data ± SD [s]
LSTM	0.171	0.031
Mantis-8M + CNN	**0.024**	**0.0078**
Mantis-8M + LSTM	0.035	0.0098

Statistics were calculated from 500 randomly selected time frames extracted from the FOI operational ECG dataset. Although the Mantis-8M + CNN and Mantis-8M + LSTM models achieved lower inference times for single ECG time frames than the LSTM model, this computational advantage should be considered alongside the substantially higher detection performance of the LSTM reported in Table 1. These findings illustrate the trade-off between inference latency and detection accuracy for real-time physiological monitoring applications. Bold values indicate the shortest mean single-frame inference time and its corresponding standard deviation.

Because training of deep neural networks involves stochastic optimization, repeated training runs using identical data and model architectures may produce small variations in model performance due to differences in random initialization and optimization trajectories. To quantify this variability, the LSTM model was retrained 100 times using different random initialization seeds while keeping all other aspects of the training procedure unchanged. Across the FOI operational ECG dataset, the retrained models achieved a mean F1-score of 0.9630 with a 95% CI of 0.9626–0.9633. The narrow CI indicates excellent reproducibility demonstrating that stochastic optimization has only a negligible effect on the final detection performance.

### 3.6 Implications for HRV analysis

Accurate HRV analysis depends on the precise estimation of inter-beat (RR) intervals, which are highly sensitive to both missed detections and false positives. Even small inconsistencies in R-peak timing can introduce distortions in time-domain and frequency-domain HRV metrics.

The high recall observed across the evaluated AI-based models indicates that missed beats are rare, reducing the risk of artificially prolonged RR intervals. Likewise, the strong precision limits the occurrence of spurious detections that would otherwise introduce artificially shortened intervals. Together, these properties support the generation of stable and physiologically consistent RR interval series.

Given the robustness of the proposed models under both benchmark and operational conditions, the results indicate that AI-based R-peak detection can provide a reliable basis for HRV computation in noisy, real-world environments without the need for manual correction of beat annotations. This conclusion is further strengthened by the substantially lower RR interval error achieved by the LSTM model, together with the narrow CIs, excellent reproducibility across repeated training runs, and consistent subject-wise performance observed across varying signal qualities. Collectively, these findings indicate that the resulting RR interval series provide a robust foundation for downstream HRV analysis in short-term recordings (Berntson et al., 1997; Shaffer and Ginsberg, 2017).

## 4 Discussion

Overall, the present study investigated whether robust, real-time AI-based R-peak detection can be achieved in noisy and operationally realistic environments. By combining physiologically consistent annotation strategies with modern machine learning architectures, the results demonstrate that high detection performance is attainable not only under controlled benchmark conditions but also in monitoring-grade ECG recordings characterized by substantial noise and signal variability. These findings extend beyond algorithmic performance, indicating that AI-based models can maintain physiologically consistent beat-to-beat timing under realistic conditions, thereby supporting reliable downstream analysis.

### 4.1 AI performance in noisy physiological data

Consistent with previous findings, the strongest-performing AI-based models, particularly the LSTM-based and Mantis-8M + CNN approaches, outperformed classical approaches across most benchmark and operational evaluation conditions (Elgendi, 2013; Hannun et al., 2019; Laitala et al., 2020). While the Pan–Tompkins algorithm maintained high recall under certain conditions, its precision degraded substantially in noisy recordings, reflecting its sensitivity to amplitude variations and non-physiological artifacts. In contrast, the LSTM-based and Mantis-8M + CNN models demonstrated strong performance across both precision and recall, indicating a greater ability to discriminate between true cardiac events and artifact-driven signal distortions (Hannun et al., 2019; Laitala et al., 2020). Importantly, the LSTM-based model demonstrated the most consistent performance across all evaluation conditions, suggesting that explicit temporal modeling of sequential dependencies provides a strong advantage for R-peak detection in noisy physiological signals. This consistency across datasets reinforces the suitability of LSTM-based architectures for real-world physiological monitoring applications.

The additional validation analyses further strengthen these findings by demonstrating that the observed performance improvements are not limited to aggregate performance metrics. The narrow CIs and minimal variability observed across repeated training runs indicate that the proposed LSTM model provides stable and reproducible detection performance despite the stochastic optimization inherent to deep neural network training. Furthermore, subject-wise analyses demonstrated that the model maintained robust performance across varying signal qualities and individual recordings, supporting its suitability for real-time physiological monitoring in operational environments.

This improvement can be attributed to the capacity of data-driven models to learn complex temporal and morphological patterns that extend beyond handcrafted features. In particular, the LSTM model leverages temporal dependencies across multiple cardiac cycles, allowing it to contextualize local signal fluctuations within a broader physiological sequence. Similarly, the foundation model approach benefits from pretrained representations that capture generalizable features across diverse time-series domains, supporting robust performance even under heterogeneous signal conditions.

Importantly, the results obtained on the FOI dataset demonstrate that these advantages persist in operationally relevant data. The ability of AI-based methods to maintain high precision and recall in this context indicates that physiologically consistent detection can be achieved despite substantial signal degradation. The strong performance observed on the FOI dataset further suggests that models trained on benchmark physiological data can generalize to monitoring-grade ECG acquired under operational conditions.

### 4.2 The role of training data: physiological validity in model learning

A central consideration in this work is the role of training data in shaping model performance. While modern AI architectures offer substantial representational capacity, their effectiveness depends on exposure to physiologically consistent patterns during training. In the context of ECG analysis, this is particularly important, as R-peak detection requires precise temporal alignment with underlying cardiac events (Berntson et al., 1997; Goldberger et al., 2000).

The substantially lower RR interval error achieved by the proposed LSTM model further suggests that improvements in R-peak detection extend beyond conventional classification metrics. Accurate preservation of beat-to-beat timing represents a prerequisite for reliable downstream physiological analyses, including HRV estimation. Although comprehensive validation of HRV metrics remains an important topic for future investigation, the improved temporal accuracy observed in the present study provides a stronger foundation for subsequent cardiovascular signal analysis and future multimodal physiological monitoring.

In addition, the annotation framework employed in this study was designed to preserve physiologically plausible timing relationships in monitoring-grade ECG recordings, while avoiding aggressive signal manipulation. By constraining corrections to annotation-level adjustments and maintaining the original signal time base, the resulting reference data provide a consistent representation of beat-to-beat cardiac dynamics.

Importantly, the role of such annotations is confined to the model development phase. Once trained, the AI-based models operate independently of manual correction, relying instead on learned representations of physiological structure. The results indicate that, when trained on physiologically consistent data, these models can generalize effectively to noisy and heterogeneous conditions without requiring access to annotated reference signals during deployment. These findings suggest that the effectiveness of AI-based R-peak detection depends primarily on the physiological validity of training data. Importantly, such validity can be achieved through a combination of expert knowledge, high-quality annotated datasets, and automated procedures, rather than through manual data cleaning alone.

### 4.3 Toward real-time physiological monitoring in operational environments

From an application perspective, the ability to perform accurate R-peak detection in real time is a key requirement for continuous monitoring of physiological state in operational contexts. In applications such as aviation, defense, and high-performance human–machine interaction, timely assessment of operator state is essential for both safety and performance optimization (Parasuraman and Wilson, 2008).

From an operational perspective, computational efficiency is an essential consideration for real-time physiological monitoring. Although the Mantis-based architectures achieved lower inference times for individual ECG frames, the proposed LSTM combined substantially higher detection performance with the shortest processing time for complete dataset inference. These findings suggest that the LSTM offers a favorable balance between detection accuracy, computational efficiency, and deployment feasibility for continuous real-time physiological monitoring in operational environments.

The findings of this study indicate that AI-based approaches are well-suited for real-time deployment, provided that models are trained on data that adequately reflect the conditions of use. The combination of monitoring-grade ECG data and physiologically valid benchmark training data enables models to maintain detection performance under realistic constraints.

Importantly, while annotation quality is critical during model development, the deployed system operates autonomously, without the need for manual intervention. This distinction is essential for practical implementation, as it separates the requirements of model training from those of real-time operation. The present work focuses on monitoring-grade ECG in operational environments, where signal quality is inherently variable and robustness is critical for downstream analysis. The objective is therefore to assess robustness and temporal consistency for HRV analysis under noise, rather than diagnostic accuracy or clinical interpretation of ECG morphology.

Accurate R-peak detection is a prerequisite for reliable HRV analysis, given the sensitivity of HRV metrics to beat-level timing errors (Berntson et al., 1997; Shaffer and Ginsberg, 2017). Improvements in detection accuracy therefore translate directly into more stable and interpretable physiological measures.

The results of this study indicate that AI-based detection can maintain physiologically consistent beat-to-beat timing even in monitoring-grade ECG recordings characterized by noise and signal variability. This supports the generation of stable RR interval series without the need for manual correction, thereby enabling direct computation of HRV metrics under operational conditions.

The relationship between recall and precision also warrants consideration when interpreting model performance in operational ECG recordings. High recall indicates that the majority of reference R-peaks are successfully detected, whereas reduced precision reflects the presence of additional predicted peaks not represented in the reference annotations. In monitoring-grade ECG recordings characterized by substantial noise and signal variability, not all detections classified as false positives necessarily reflect algorithmic failure. Some detections may correspond to physiologically plausible cardiac events that were not included in the reference annotations because of annotation uncertainty in heavily contaminated signal segments. Conversely, other detections may represent genuine false positives arising from artifact contamination, duplicate detections, or clustered predictions occurring near the same cardiac event. Further inspection of such cases may therefore provide additional insight into both annotation uncertainty and model behavior under noisy conditions.

In addition, some predicted peaks were occasionally observed in close temporal proximity. Introducing post-processing constraints, such as a physiologically informed minimum inter-peak distance, may further improve precision by suppressing temporally implausible duplicate detections while preserving true cardiac events.

This capability is particularly relevant in real-time applications, where physiological signals are used to infer cognitive state dynamically. By providing reliable input to HRV analysis, the proposed approach contributes to the development of adaptive human–machine systems capable of responding to operator state in real time.

### 4.4 Foundation models and generalization across domains

The inclusion of a foundation model for time-series analysis highlights the potential for transfer learning in physiological signal processing. While LSTM-based models demonstrated the strongest and most consistent performance across datasets, the results obtained with the Mantis-8M architecture suggest that pretrained representations can also support robust detection when appropriately adapted to the task (Zerveas et al., 2021).

However, the performance of foundation models remains dependent on the alignment between pretraining data and the target domain. In the context of ECG analysis, this underscores the importance of domain-specific adaptation to ensure that learned representations reflect physiologically consistent patterns. When appropriately aligned, such models can support robust detection across diverse signal conditions.

These findings indicate that future developments in this area may benefit from combining large-scale pretraining with physiologically grounded validation frameworks, enabling both scalability and reliability in physiological AI systems.

One possible explanation for the reduced performance observed in the Mantis-8M + LSTM configuration is that the pretrained Mantis-8M architecture primarily functions as a feature extractor rather than a task-specific sequential detector. While the extracted representations contain rich temporal information, additional investigation may be required to determine how such features are best adapted for point-wise R-peak classification. Future work may therefore benefit from optimizing the downstream architecture or integrating task-specific training strategies more directly into the pretrained backbone.

Foundation models are often motivated by their potential to support transfer learning and reduce the amount of domain-specific data required for adaptation. However, the strong performance and generalization capability demonstrated by the LSTM-based model on the FOI operational dataset suggest that the temporal structure of ECG signals may be sufficiently consistent across subjects and noise conditions to enable robust inference without domain-specific retraining. Under the present conditions, this reduced the expected advantage of the foundation-model approach and highlights the effectiveness of physiologically grounded sequential modeling for operational ECG analysis.

## 5 Limitations

Several limitations should be considered. First, the FOI dataset, while operationally relevant, represents a limited sample size and may not capture the full variability of real-world conditions. Second, the evaluation focuses on R-peak detection accuracy and does not directly quantify downstream improvements in HRV metrics or cognitive state classification. Third, while the models are designed for real-time use, computational performance and latency were not explicitly benchmarked in this study.

A further limitation relates to the reference annotations used for evaluation. Although the annotation framework was designed to maximize temporal consistency and physiological plausibility, reference annotations in monitoring-grade ECG recordings should not be interpreted as infallible representations of cardiac activity, particularly in heavily contaminated signal segments. Consequently, some detections classified as false positives may correspond to physiologically plausible cardiac events that were not included in the reference annotations, while others may represent genuine false-positive detections arising from artifact contamination or signal ambiguity. This limitation is inherent to the evaluation of noisy operational ECG recordings and should be considered when interpreting performance metrics obtained under real-world conditions. Future work should investigate the impact of annotation uncertainty on model performance and explore strategies for uncertainty-aware inference.

The study does not include clinical-grade ECG data or diagnostic evaluation; therefore, the conclusions are limited to monitoring and operational use cases.

## 6 Future directions

Future research should focus on extending the present framework toward fully integrated physiological monitoring systems capable of continuous real-time operation in operational environments. This includes (i) evaluating model performance during continuous real-time deployment, (ii) quantifying the impact of improved R-peak detection on downstream HRV and cognitive state models, and (iii) integrating additional physiological modalities, such as respiration and eye-tracking, to support multimodal state estimation.

An additional priority is to enable interoperability with established physiological signal analysis frameworks (e.g., NeuroKit2) for standardized downstream HRV computation and multimodal physiological analysis. Such integration would combine robust AI-based R-peak detection under challenging operational conditions with well-established physiological signal analysis toolkits, providing a scalable foundation for future real-time physiological monitoring systems.

The strong generalization performance observed in the LSTM-based model further suggests that future work should investigate how physiologically grounded sequential models scale across broader operational populations, sensor configurations, and noise conditions. Systematic ablation studies should also be performed to quantify the relative contributions of training data augmentation, model architecture, and annotation methodology to overall detection performance. In parallel, additional research into foundation models for physiological data remains warranted, particularly regarding domain adaptation, transfer learning, and task-specific downstream architectures for ECG analysis.

Finally, the establishment of standardized datasets, annotation frameworks, and evaluation protocols for monitoring-grade ECG would facilitate more consistent benchmarking and accelerate the development of reliable AI-based physiological monitoring systems.

## 7 Conclusion

In summary, this study demonstrates that robust AI-based R-peak detection can be achieved in noisy, operationally realistic ECG environments using physiologically valid benchmark training data. Across benchmark, noise-augmented, and real-world operational datasets, the strongest-performing evaluated models maintained high precision and recall despite substantial signal variability and artifact contamination.

Among the evaluated approaches, the LSTM-based model demonstrated the most robust and consistent performance, particularly on monitoring-grade ECG recordings acquired under operational conditions. The additional statistical validation, including CI estimation, subject-wise performance analyses, and reproducibility across repeated training runs, further supports the stability and robustness of the proposed approach. Importantly, the strong performance observed on the FOI dataset indicates that physiologically grounded sequential models can generalize effectively to noisy operational data without requiring domain-specific retraining or manual intervention during deployment.

The substantially lower RR interval error achieved by the proposed LSTM model further demonstrates improved preservation of physiologically consistent beat-to-beat timing under monitoring-grade conditions. These findings provide a robust foundation for downstream HRV analysis while recognizing that comprehensive validation using HRV metrics derived from different detection methods remains an important direction for future work.

Taken together, these results support the feasibility of AI-based ECG analysis as a reliable foundation for real-time physiological and cognitive state monitoring in complex operational environments.

## Data Availability

The raw data supporting the conclusions of this article will be made available by the authors, without undue reservation.

## Footnotes

1 The Python package “py-ecg-detectors” contains a ready-to-use Pan−Tompkins algorithm for R-peak detection. Available at: https://pypi.org/project/py-ecg-detectors/ (accessed May 12, 2026).

2 The “Complete Pan−Tompkins Implementation ECG QRS detector” is an algorithm developed by Hooman Sedghamiz (April 8, 2018). Available at: https://se.mathworks.com/matlabcentral/fileexchange/45840-complete-pan-tompkins-implementation-ecg-qrs-detector (accessed May 12, 2026).
